# Wanted

**Published:** 1889-01

**Authors:** 


WE KNOW OF NO MORE SUBSTANTIAL WAY in which to present our readers the greeting of the New Year, nor any more fitting manner in which to recognize our appreciation of generous support by our subscribers, whose number is constantly increasing, than by presenting them with a JOURNAL so laden with "good things" that it takes *thirty-two* extra pages to hold them all. The February number, too, will be full of interest. Among other interesting features, it will contain the first of a series of articles from the pen of Dr. Edward M. Moore, of Rochester, on The Surgery of the Upper Extremities. It will be a real pleasure to his many friends throughout this country and Europe to see his name again in our columns as a contributor, where he always has an admiring and appreciative audience.

---

WANTED.—A few copies of this journal for January, 1887, for which the usual price of single copies, twenty-five cents apiece, will be paid. Address THE BUFFALO MEDICAL AND SURGICAL JOURNAL, 284 Franklin street, Buffalo, N. Y.

---

LEE'S METALLIC SPLINTS (adv., page xxvii.), are a serviceable invention for injuries of the forearm. They combine, with several good surgical points, the element of cheapness, which is of considerable importance. They can be obtained of the manufacturers, at Conshohocken, Pa.

WE ALWAYS HAD an exalted opinion of the varied accomplishments of the editor of *Progress,* but we had no idea he was clever enough to persuade such a staunch teetotaler as Dr. Wathen to eat his punch pineapple even unto helplessness. "Can such things be, and overcome us like a summer's cloud, without our special wonder?"

